# Interactions of Seedborne Bacterial Pathogens with Host and Non-Host Plants in Relation to Seed Infestation and Seedling Transmission

**DOI:** 10.1371/journal.pone.0099215

**Published:** 2014-06-17

**Authors:** Bhabesh Dutta, Ronald Gitaitis, Samuel Smith, David Langston

**Affiliations:** Department of Plant Pathology, University of Georgia, Coastal Plain Experiment Station, Tifton, Georgia, United States of America; University of the West of England, United Kingdom

## Abstract

The ability of seed-borne bacterial pathogens (Acidovorax citrulli, Clavibacter michiganensis subsp. michiganensis, Pseudomonas syringae pv. tomato, Xanthomonas euvesicatoria, and Pseudomonas syringae pv. glycinea) to infest seeds of host and non-host plants (watermelon, tomato, pepper, and soybean) and subsequent pathogen transmission to seedlings was investigated. A non-pathogenic, pigmented strain of Serratia marcescens was also included to assess a null-interacting situation with the same plant species. Flowers of host and non-host plants were inoculated with 1×10^6^ colony forming units (CFUs)/flower for each bacterial species and allowed to develop into fruits or umbels (in case of onion). Seeds harvested from each host/non-host bacterial species combination were assayed for respective bacteria by plating on semi-selective media. Additionally, seedlots for each host/non-host bacterial species combination were also assayed for pathogen transmission by seedling grow-out (SGO) assays under greenhouse conditions. The mean percentage of seedlots infested with compatible and incompatible pathogens was 31.7 and 30.9% (by plating), respectively and they were not significantly different (P = 0.67). The percentage of seedlots infested with null-interacting bacterial species was 16.8% (by plating) and it was significantly lower than the infested lots generated with compatible and incompatible bacterial pathogens (P = 0.03). None of the seedlots with incompatible/null-interacting bacteria developed symptoms on seedlings; however, when seedlings were assayed for epiphytic bacterial presence, 19.5 and 9.4% of the lots were positive, respectively. These results indicate that the seeds of non-host plants can become infested with incompatible and null-interacting bacterial species through flower colonization and they can be transmitted via epiphytic colonization of seedlings. In addition, it was also observed that flowers and seeds of non-host plants can be colonized by compatible/incompatible/null-interacting bacteria to higher populations; however, the level of colonization differed significantly depending on the type of bacterial species used.

## Introduction

Seedborne phytopathogenic bacteria act as primary inoculum source for many important vegetable diseases. Examples of these include watermelon fruit blotch [1], [4], [23], [33], [47], [48], [50], bacterial canker of tomato [21], [43], bacterial speck of tomato [30], bacterial spot of pepper [26], [27], [42], and bacterial blight of soybean [28], which are caused by *Acidovorax citrulli* [35], [38], *Clavibacter michiganensis* subsp. *michiganensis* (Smith) [43], *Pseudomonas syringae* pv. *tomato* (Okabe) Young, Dye & Wilkie, *Xanthomonas euvesicatoria* [27] and *Pseudomonas syringae* pv. *glycinea* (Coerper) Young, Dye and Wilkie, respectively. These bacterial diseases are economically important to their respective hosts and in most cases, infested seeds and seedlings serve as a primary inoculum source for epidemics in the greenhouse and in the field [20]. However, primary inoculum can also arise from other sources like volunteer plants [25], [44], weeds [19], [36], intact plant debris [2], [13], infected transplants [18], insects [6], [51] and contaminated tools and equipment [16], [43]. Nevertheless, seedborne inoculum remains a matter of paramount importance for most of these diseases. As a precautionary step, many of the major vegetable seed producers have moved their seed production to areas of the world that are less conducive to bacterial diseases, but seedborne bacterial diseases still cause millions of dollars in losses annually [5], [20].

Seeds are passive carriers of plant pathogens across geo-political borders and may even be responsible for the introduction of exotic diseases to new areas or sometimes may also account for reemergence of past diseases [20]. Seedborne bacterial pathogens are of major concern as strategies to manage bacterial disease are limited and often ineffective [20]. Seed health testing and seed treatments are regularly used to exclude seedborne bacterial inoculum [20]. In addition, use of pathogen-free seed is recommended to manage these diseases and as a result seedlots are routinely tested for bacterial pathogens by the regulatory agencies and seed companies. Some of the important seed health assays include seedling grow-out (greenhouse), sweat-box test, recovery on semi-selective medium, enzyme linked immunosorbent assay (ELISA), and polymerase chain reactions (conventional, real-time) [9], [20], [29]. Despite these measures, epidemics due to seedborne inoculum continue to occur, although sporadically. Hence, a better knowledge of seed infestation/infection process by seedborne bacteria is required to prevent introduction and exclusion of seedborne inoculum.

Among the various pathways of seed infection, the floral pathway of seed infection has been shown to be permissive especially for plant pathogenic bacteria [9], [45], [49]. This pathway of seed infection has been reported in many plant pathogenic bacterial pathosystems including *A. citrulli*-watermelon, *Xanthomonas campestris* pv. *campestris*-cauliflower, and *X. euvesicatoria*-pepper [9], [45], [49]. Although, seed infestation and seedling transmission process have been demonstrated with seedlots developed from flower inoculations, the majority of these studies were conducted using compatible situations (host-corresponding plant pathogenic bacteria). In a significant development, Darrasse *et al*. (2010) [7] observed that inoculation of bean flowers with *X. campestris* pv. *campestris* resulted in a high level of infested bean seeds (an incompatible interaction). Interestingly, *X. campestris* pv. *campestris* was also transmitted efficiently to bean seedlings (incompatible situation). As expected, the inoculation of bean flowers with a compatible pathogen (*X. citri* pv. *phaseoli* var. *fuscans* (a compatible interaction)) resulted in efficient seed infestation and seedling transmission of pathogen. Irrespective of interactions tested, population dynamics of bacteria were similar on bean seedlings. These results suggest a probable role of seed of a non-host plant to serve as an inoculum source for seedborne plant pathogenic bacteria. In addition, the authors clearly demonstrated that the flowers of a non-host plant can result in infested seeds upon inoculation with an incompatible pathogen [7]. Recently, it was observed that *A. citrulli* can also efficiently colonize pepper flowers and could result in infestation of pepper seeds [11]. However, the transmission of *A. citrulli* in pepper seedlings was not evaluated as in the above study.

In this manuscript, we investigated four important steps in the seed transmission process through flowers; namely, flower colonization, seed infestation, seed colonization and seedling transmission of five different seedborne bacterial pathogens (*A. citrulli*, *C. michiganensis* subsp. *michiganensis*, *P. syringae* pv. *tomato*, *X*. *euvesicatoria*, and *P. syringae* pv. *glycinea*) with their respective compatible and incompatible hosts (watermelon, tomato, pepper, and soybean). In addition, the above parameters were also assessed for an incompatible situation, a non-host plant (onion) vs. the seedborne bacterial pathogens stated above. For a null-interacting situation, a non-pathogenic pigmented strain of *Serratia marcescens* was also included in this study.

## Materials and Methods

### Bacterial strains and inoculum preparation

Bacterial strains of *A. citrulli*, *C. michiganensis* subsp. *michiganensis*, *P. syringae* pv. *tomato*, *X*. *euvesicatoria*, *P. syringae* pv. *glycinea*, and *Serratia marcescens* (a non-pathogenic, red-colored strain) used in this study are described in Table 1. Bacterial strains were routinely grown on yeast extract-dextrose–CaCO3 (YDC), nutrient agar (NA) media (Becton-Dickinson, Sparks, MD) or King's medium B (KMB) for 48-h at 28°C. The identities of bacterial strains were confirmed by respective species specific PCR assay [3,22,31,52,53]. In addition, physiological and pathogenicity tests diagnostic for bacterial identifications were also conducted [37].

**Table 1:** Identity, origin, and source of bacterial strains used in this study.

Bacterium	Strain^1^	Host	Tissue	Reference
*Xanthomonas euvesicatoria*	XCV 04-100	Pepper	Leaves	[9]
*Acidovorax citrulli*	AAC 00-1	Watermelon	Leaves	[48]
*Pseudomonas syringae pv. tomato*	PST 89-43	Tomato	Leaves	This study
*Clavibacter michiganensis* subsp. *michiganensis*	CMM 97-1	Tomato	Leaves	This study
*Pseudomonas syringae* pv. *glycinea*	PSG 86-3	Soybean	Leaves	This study
*Serratia marcescens*	SM 12-1	Onion	Bulb	This study

1 R.D. Gitaitis, CPES-UGA Bacterial Culture Collection.

Inoculum was prepared by transferring single colonies of each bacterial strain from 48-h old cultures on NA (*A. citrulli*, *C. michiganensis* subsp. *michiganensis*, and *S. marcescens*), YDC (*X*. *euvesicatoria*) or KMB (*P. syringae* pv. *tomato* and *P. syringae* pv. *glycinea*) media to nutrient broth. This was followed by overnight incubation at 28°C on a rotary shaker (Innova; New Brunswick Scientific Co., Edison, NJ) at 150 rpm. After overnight incubation, bacterial cultures were centrifuged at 5,000×g (Allegra 25R, Beckman Coulter, Fullerton, CA) for 3 min and the supernatant was decanted leaving a pellet of bacterial cells. The pellet was resuspended in 0.1 M phosphate (KH_2_PO_4_ – K_2_HPO_4_) buffered saline (0.75% NaCl) solution at pH 7.0 (PBS) and the concentration of bacterial cells was adjusted using a spectrophotometer (Spectronic 20; Bausch and Lomb, Rochester, NY) to an optical density of 0.3 at 600 nm (≈1×10^8^ colony forming units (CFU)/ml). This was serially diluted in PBS to obtain the desired concentration according to each experiment.

### Confirmation of compatible/incompatible/null-interaction with bacterial strains on different host and non-host plants

In order to confirm the type of interactions with different bacterial species and plants used in this study, a 1 ml of bacterial suspension containing 1×10^8^ CFU/ml was injected with hypodermic needle into the leaves of 3-to-4 wk old watermelon (cv. Crimson sweet), tomato (cv. Roma), pepper (cv. Aristotle), soybean (cv. AG4831, Asgrow), and onion (cv. Century) plants under greenhouse conditions of 28°C and 70% RH. The production of symptoms were determined 48-h to 72-h after inoculation to confirm that the interactions of bacterial strains with their respective hosts were compatible. In contrast, production of hypersensitive reactions (localized cell death) on plant species (other than their respective compatible hosts) after 12-h to 24-h of inoculation confirmed that bacterial strains were incompatible. However, failure to produce disease symptoms or hypersensitive responses on any of the plant species used was considered a null-interaction as used in other studies with *Escherichia coli* [7], [8].

### Effect of flower inoculation on seed infestation of host and non-host plants by bacterial species

*Acidovorax citrulli*, *C. michiganensis* subsp. *michiganensis*, *P. syringae* pv. *tomato*, *X*. *euvesicatoria*, *P. syringae* pv. *glycinea*, and *S. marcescens* were cross-inoculated on to the flowers of watermelon, tomato, pepper, soybean, and onion. All infested seedlots, except for onion, were produced under greenhouse conditions. Onions were produced in the field. In two independent trials, 50 plants each of watermelon, tomato, pepper and soybean were grown in 10-L plastic pots containing commercial potting mix (Scotts Miracle-Gro Company, Marysville, OH). Pots were spaced evenly on benches under greenhouse conditions of 25° to 30°C, a mean RH of 76%, and 12-h of natural light daily. Plants were maintained under greenhouse conditions for approximately 90-120 days. At anthesis, 10 µl of inoculum containing ∼1×10^8^ *A. citrulli* or *C. michiganensis* subsp. *michiganensis* or *P. syringae* pv. *tomato* or *X*. *euvesicatoria* or *P. syringae* pv. *glycinea* or *S. marcescens* CFU/ml (∼1×10^6^ CFU/flower) were deposited on to the stigmata of flowers. At least ten flowers per plant per bacterial species were inoculated and left to maturate for 40–45 days. Fruit were harvested at maturity and seeds were extracted manually. Seedlots generated by treating flowers of tested plant species with 0.1 M PBS served as negative controls. For seed extraction, fruits were surface sterilized by wiping with 70% ethanol and seeds were excised with a sterilized knife. Seeds were air-dried in sterile petri dishes with partially open-lids under a laminar air flow chamber for 72-h. Seeds from individual fruit were maintained as a separate seedlot and stored in paper bags at 4°C.

Onion seedlings with intact roots were kindly provided by the Vidalia Onion and Vegetable Research Center (VOVRC), Reidsville, GA which were transplanted in field plots at the Black Shank Farm near Tifton, GA on November 28, 2012. The seedlings were planted in raised, beds with plant spacing between seedlings of ∼15 cm. For flower inoculation, stigmata were spray inoculated with a 10 ml of bacterial suspension containing ≈1×10^5^ CFU/ml (≈1×10^6^ CFU/flower). After treatment, flower heads were covered with a plastic cover and incubated for 24-h. After a period of incubation, plastic covers were removed and flower heads were allowed to mature. Onion flower heads treated with 0.1 M PBS in a similar manner served as a negative control. After 60 days post inoculation (DPI), onion seed heads were harvested and seeds from heads were manually extracted. Seeds harvested from each flower head were maintained as a separate lot. Seedlots were stored in plastic bags at 4°C until they were processed.

Samples of seedlots [*n* = 5 g (pepper (800 seeds), soybean (100 seeds), watermelon (100 seeds), tomato (500 seeds), onion (1000 seeds)) /lot/bacterial strain] in three replicates were placed separately in extraction bags (Bioreba, Reinach, Switzerland) and macerated using a tissue homogenizer (Homex 6, Bioreba) followed by the addition of 10 ml 0.1 M PBS. The resulting suspension in each bag was agitated by gentle shaking for one minute and transferred to 50-ml polypropylene screw cap tubes. The suspension was vortexed for 30 s followed by centrifugation at 8000×g for 5 min. Subsequently, the supernatant was decanted and the pellet was resuspended in 1 ml 0.1 M PBS. Ten-fold serial dilutions were prepared in 0.1 M PBS, from which aliquots of 0.1 ml were spread-plated onto respective semi-selective media. The semi-selective media used for *A. citrulli, C. michiganensis* subsp. *michiganensis*, *P. syringae* pv. *tomato*, *X*. *euvesicatoria*, and *P. syringae* pv. *glycinea* were Numhem's medium (a proprietary medium), mCNS [17], KFT [39], Tween B [40], and BNQ [37] medium, respectively. For *S. marcescens*, SD [14] medium was used for isolation. After incubation at 28°C for 24 to 48-h, presence of *A. citrulli, C. michiganensis* subsp. *michiganensis, P. syringae* pv. *tomato, X*. *euvesicatoria, P. syringae* pv. *glycinea,* or *S. marcescens* colonies from each seed macerate was determined on plates. Additional confirmation of the identity of the bacterial colonies was conducted by a real-time PCR assay using species-specific primers [3,22,31,52,53]. Identity of colonies was also confirmed by different diagnostic physiological and pathogenicity tests [37]. Seed samples from 10 negative control lots from each plant species were processed similarly. A seedlot was considered to be positive when respective bacterial species was detected on agar plates from any of the three replicates. The percentage- of *A. citrulli*, *C. michiganensis* subsp. *michiganensis*, *P. syringae* pv. *tomato*, *X*. *euvesicatoria, P. syringae* pv. *glycinea*, and *S. marcescens* positive lots detected by plating was determined.

### Seed-to-seedling transmission of phytobacteria to host and non-host plants

Seedlots generated previously by flower inoculation were tested for transmission of phytobacterial species from the seeds of host and non-host plants. Seed samples [*n* = 5 g (pepper (800 seeds), soybean (100 seeds), watermelon (100 seeds), tomato (500 seeds), onion (1000 seeds))/lot/phytobacterial species/host or non-host plant] in three replicates were tested for *A. citrulli*, *C. michiganensis* subsp. *michiganensis*, *P. syringae* pv. *tomato*, *X*. *euvesicatoria*, *P. syringae* pv. *glycinea*, and *S. marcescens* transmission by seedling grow out (SGO) assay. For SGO, seed samples were planted in 128-cell speedling trays containing commercial potting mix and placed contiguously under greenhouse conditions at 28°C and >85% R.H. In order to reduce the risk of cross-contamination among treatments from splash dispersal of bacteria, careful manual watering (rather than automated overhead irrigation) was used. At 18 DPI, seedlings were scored for disease symptoms. Seed samples from 10 negative control lots for each host/non-host plant were evaluated as described above. The mean percentage of infested seedlots [(number of seedlots with at least one symptomatic seedling)/(total number of seedlots) ×100], as well as the mean percentage disease transmission within individual seedlot [(number of symptomatic seedlings per lot)/(total number of germinated seedlings per lot) ×100] were calculated. In order to confirm the identity of the observed putative symptoms on seedlings, compatible pathogens were recovered from at least two symptomatic seedlings from each of the positive lots. For each tested seedlings, a small (∼2-mm^2^) piece of cotyledon tissue along the lesion margin was cut using sterile forceps and macerated in 100 µl of sterile 0.1 M PBS. A loopful of tissue macerates was spread-plated onto semi-selective agar medium and incubated for 48-h at 28°C. Putative bacterial colonies grown on the plates were tested using a species-specific real-time PCR assay [3,22,31,52,53]. A seedlot was considered to be positive when at least one seedling developed symptoms from any of the three replicates tested. The percentage of positive lots for each host/non-host bacterial species combinations was calculated. Additionally, disease transmission percentages from individual lots were also calculated for each host/non-host bacterial species.

Seedlots that did not produce any symptoms 18 DPI were assessed for epiphytic bacterial presence by dilution plating of a seedling wash on respective semi-selective media. Briefly, 10 seedlings per replicate per host/non-host bacterial species combinations were removed from speedling trays and roots were excised using sterile scissors. The remaining seedlings were placed in 100 ml of PBS in a 250 ml conical flask and shaken overnight at approximately 150 rpm on a rotary shaker at 25°C. After incubation, a 1.0 ml aliquot was removed and spread-plated on respective semi-selective medium. After 24-48 h of incubation, bacterial colonies were tentatively identified as *A. citrulli* or *C. michiganensis* subsp. *michiganensis* or *P. syringae* pv. *tomato* or *X*. *euvesicatoria* or *P. syringae* pv. *glycinea* or *S. marcescens*. Bacterial identities were confirmed using a species-specific PCR assay as described above. A seedlot was considered to be positive when the target bacterium was recovered from any of the three replicates tested. The mean percentage bacterial transmission for host/non-host bacterial species combinations were calculated as [(number of positive lots)/(total number of lots tested) ×100]. In addition, germination percentages of seedlings for each host/non-host bacterial species combinations were also recorded.

### Confirmation of bacterial strains re-isolated from infested seedlots (plating and SGO) by repetitive extragenic palindrome (rep)-PCR

Strains isolated from each seedlot (from plating and SGO assay) were inoculated into 3 ml of nutrient broth and incubated on a rotary shaker (Innova; New Brunswick Scientific Co., Edison, NJ) at 250 rpm for 18-h. After incubation, cells were harvested by centrifugation at 6,000×g (Allegra 25R, Beckman Coulter, Fullerton, CA) for 5 min and DNA was extracted using the UltraClean Microbial DNA Kit (MO BIO, Carlsbad, CA) according to the manufacturer's instructions. For rep-PCR, two microliters of bacterial DNA from each isolate (5 ng/µl) were amplified in 23 µl of PCR master mix containing 10 mM Tris-HCl (pH 9.0), 50 mM KCl, 0.1% Triton X-100, 1.5 mM MgCl_2_, 0.2 mM of each nucleotide (dATP, dCTP, dGTP, and dTTP), 10µM of BOXA1R primer (5′-CTA CGG CAA GGC GAC GCT GAC G-3′) [46], and 1 unit of Taq DNA polymerase (Promega, Madison, WI) per reaction. DNA amplification was carried out in a Mastercycler Gradient programmable thermal cycler (Eppendorf, Hamburg, Germany). The PCR amplification protocol included an initial denaturation at 95°C for 7 min followed by 30 cycles of denaturation at 95°C for 1 min, annealing at 53°C for 1min, and extension at 65°C for 8 min. PCR products (10 µl) were separated by electrophoresis at 125 V for 4-h on a 1.5% agarose gel in 1X Trisborate ethylenediaminetetraacetic acid (EDTA) buffer. For comparison, rep-PCR was also conducted on DNA extracted from reference bacterial strains, which were used for flower inoculations. DNA fingerprinting patterns of isolated bacterial strains from seedlots were compared with the patterns of their reference strain.

### Temporal bacterial population dynamics on flowers of host and non-host plants

The temporal population dynamics of *A. citrulli*, *C. michiganensis* subsp. *michiganensis*, *P. syringae* pv. *tomato*, *X*. *euvesicatoria*, *P. syringae* pv. *glycinea*, and *S. marcescens* on watermelon, tomato, pepper, and soybean flowers were investigated under greenhouse conditions. Twenty five mature plants for each host/non-host bacterial species combination were established under greenhouse conditions and maintained as described above. Stigmata of flowers were inoculated with a 10 µl suspension containing 1×10^4^ CFU/ml (∼1×10^2^ CFU/flower) of each bacterial species as described above. Twenty flowers per plant per bacterial species were used for this experiment. At each sampling period (0, 6, 12, 24, 48, 96 hours post inoculation (hpi)), three inoculated flowers were harvested and processed as follows. After harvest, petals were removed and stigma and style (together) were excised carefully using a sterile scalpel. The excised tissues were placed in 1.5 ml micro-centrifuge tubes which were then macerated with a disposable plastic pestle (ACT Gene AgileGrinder portable disposable plastic pestle, Thomas Scientific, Swedesboro, NJ). Tissue macerates were suspended in 1 ml of PBS, vortexed vigorously for 1 min following by 10-fold serial dilutions in 1 ml of PBS. Subsequently, 100 µl aliquants were spread plated on to respective semi-selective media and incubated for 24–48 h at 28°C. After incubation, bacterial colonies on respective semi-selective media were enumerated and recorded from each treated flower at each sampling point. Flowers treated with PBS and processed as described above served as a negative control.

The experiment was repeated twice and plots of log_10_ of *A. citrulli*, *C. michiganensis* subsp. *michiganensis*, *P. syringae* pv. *tomato*, *X*. *euvesicatoria*, *P. syringae* pv. *glycinea*, and *S. marcescens* CFU/flower against time were generated for each bacteria-host and non-host plants combinations and area under the growth progress curve (AUGPC) were calculated as follows: ∑*_i_*_ = 1_ [(*Y_i_*_+*n*1_ +Y*_i_*)]/2 [*X_i_*_+1_-X*_i_*] where *Y_i_* =  bacterial population at the *i*th observation, *X_i_* =  time in hours at the *i*th observation and n  =  the total number of observations. Analysis of variance was conducted on the AUGPC values to determine significance of treatment effects on flower colonization, and Fischer's Least Significant Difference (LSD) test (*P* < 0.05) was used for mean separation.

### Colonization of bacteria on host and non-host seeds

Since bacterial populations in naturally infested seeds vary widely and follow log-normal distribution [10], and also the fact that a standardized initial population is required for the bacterial colonization study, artificially infested seeds were used to conduct this experiment. Six phytobacterial species (*A. citrulli*, *C. michiganensis* subsp. *michiganensis*, *P. syringae* pv. *tomato*, *X*. *euvesicatoria*, *P. syringae* pv. *glycinea*, and *S. marcescens*) selected were inoculated separately onto host and non-host seeds (watermelon, tomato, pepper, soybean, and onion) by vacuum infiltration as described below. Seeds [number of seeds per sample (*n*)  =  15 g (pepper (2400 seeds), soybean (300 seeds), watermelon (300 seeds), tomato (1500 seeds), onion (3000 seeds))/lot/phytobacterial species/host or non-host plant] in three replicates were placed in a sterile 250 ml side-arm flask containing 20 ml of each bacterial cell suspension (∼1 10^6^ CFU/ml). Bacterial suspensions were infiltrated into the seeds (in bulk) by applying vacuum to the flask for 20 min after which flasks were subjected to normal atmospheric pressure thereby allowing the bacterial suspension to be drawn into the seeds to replace the removed air. After vacuum infiltration, seeds were drained using cheese cloth and air-dried on sterile paper towel for 24-h at 24°C. Seeds were incubated in plastic boxes (Tri-state plastics, Dixon KY) on moist blotter paper (Hoffman Manufacturing, Albany, OR) at 28°C with 100% RH and continuous fluorescent light. After 0, 6, 12, 24 and 481hpi, seed samples (*n* =  1 g (pepper (150 seeds), soybean (20 seeds), watermelon (20 seeds), tomato (100 seeds), onion (200 seeds))/replicate) were macerated using a tissue homogenizer (Homex 6, Bioreba, Reinach, Switzerland). Seed macerates were suspended in 10 ml PBS and transferred to 50 ml polypropylene screw cap tubes (Corning Inc., Tewksbury MA). Macerates were agitated vigorously at 100 rpm (Fisher Vortex Genie2; Fisher Scientific International) for 30 s and centrifuged at 8000×g (Allegra 25R centrifuge, Beckman Coulter Inc., Pasadena, CA) for 5 min. Subsequently, the supernatant was decanted and the pellet was suspended in 1 ml PBS. Ten-fold serial dilutions (1∶9) were made in PBS and aliquants of 100 µl of seed macerates were spread onto respective semi-selective agar media. After 24 to 48-h of incubation at 28°C, bacterial colonies were enumerated. The experiment was repeated two times and mean bacterial populations for each host/non-host bacterial species combinations were plotted over time to generate an AUGPC. Analysis of variance was conducted on the AUGPC values to determine significance of treatment effects on seed colonization, and Fischer's Least Significant Difference (LSD) test (*P*<0.05) was used for mean separation.

## Results

### Confirmation of compatible/incompatible/null-interaction with bacterial strains on different host and non-host plants

*A. citrulli*, *X* **.** *euvesicatoria*, and *P. syringae* pv. *glycinea* produced disease symptoms on watermelon, pepper, and soybean leaves, respectively after 72-h of inoculation. Also, *C. michiganensis* subsp. *michiganensis* and *P. syringae* pv. *tomato* produced disease symptoms on tomato leaves after 72-h of inoculation. Leaves inoculated with PBS remained asymptomatic. Inoculation of *A. citrulli*, *X*. *euvesicatoria*, *P. syringae* pv. *glycinea*, and *P. syringae* pv. *tomato* on to respective non-host plants including onion produced hypersensitive reactions after 24-h of inoculation. The hypersensitive reactions produced by *C. michiganensis* subsp. *michiganensis* were not as distinctive as produced by other bacterial species in incompatible reactions. However, after monitoring *C. michiganensis* subsp. *michiganensis* populations over 24-h post inoculation, it was observed that bacterial populations were three-fold lower than a compatible pathogens on a particular incompatible host of *C. michiganensis* subsp. *michiganensis* indicating a hypersensitive response. In contrast, inoculation of *S. marcescens* on to any of the plant species tested did not produce disease symptoms or hypersensitive reactions confirming null-interaction.

### Effect of flower inoculation on seed infestation of host and non-host plants by bacterial species

Colonies of target bacterial species were not isolated were not isolated from the seedlots of any plant species whose flowers were treated with PBS. In two separate greenhouse trials, a total of 416, 1838, and 406 fruits were produced from the flowers that were treated with compatible, incompatible and null-interacting bacterial species, respectively. Disease symptoms were not observed on fruits that were developed from compatible, incompatible and null-interacting bacterial species. However, the percentage of seedlots infested with compatible and incompatible pathogens was 31.7 and 30.9%, respectively and they were not significantly different (P = 0.67) (Table 2) as assessed by bacterial plating. The percentage of seedlots infested with null-interacting bacterial species was 16.8% and it was significantly lower than the infested lots generated with compatible and incompatible bacterial pathogens (*P* = 0.03) (Table 2). One hundred percent of the suspect bacterial colonies were identified to their respective species using species-specific real-time PCR and physiological assays.

**Table 2:** Summarized table of percentage of positive seedlots with compatible, incompatible and null-interacting bacterial species developed in fruits upon flower inoculation.

1Type of seed health assay	2Type of interactions with host or non-host plants		
	Compatible	Incompatible	Null
3Plating	31.7a	30.9a	16.8b
4Symptomatic transmission	22.4A	0B	0B
5Asymptomatic transmission	44.6*a*	19.5*b*	9.4*c*

1 *Acidovorax citrulli*, *C. michiganensis* subsp. *michiganensis*, *P. syringae* pv. *tomato*, *X*. *euvesicatoria*, *P. syringae* pv. *glycinea* and *Serratia marcescens* were cross-inoculated on to the flowers of watermelon, tomato, pepper, soybean and onion plants. Seeds developed in inoculated fruits were harvested and tested for the presence of target bacteria by plating on semi-selective medium or seedling grow out (SGO) assay.

2 Type of interaction of bacterial species with their host and non-host plants as determined by foliar infiltration of 1×10^8^ colony forming units/ml.

3 Mean percentage of seedlots tested positive for compatible, incompatible and null-interacting bacterial species by plating. Means with similar letters are not significantly different according to LSD in SAS (*P*<0.05).

4 Mean percentage of seedlots tested positive by symptomatic transmission of compatible, incompatible and null-interacting bacterial species. A seedlot was considered to be positive when a seedling in a tray showed symptoms from any of the three replicates tested. Means with similar letters are not significantly different according to LSD in SAS (*P*<0.05).

5 Mean percentage of seedlots tested positive for asymptomatic (epiphytic) transmission of compatible, incompatible and null-interacting bacterial species to developing seedlings. A seedlot was considered to be positive when the target bacterium was recovered from any of the three replicates tested. Means with similar letters are not significantly different according to LSD in SAS (*P*<0.05).

### Seed-to-seedling transmission of phytobacteria to host and non-host plants

Seedlings developed from seeds of control seedlot did not produce any symptoms at 18 DPI. Plating of seedling wash on any of the semi-selective medium did not result in bacterial growth. The mean percentage of seedlots transmitted disease to seedlings with compatible pathogens was 22.4% (range: 12.5%–36.3%) (Table 2 and Table S1). However, compatible pathogens were also detected in 44.6% (24.2%–74.8%; Table S1) of the seedlots as epiphytes on asymptomatic seedlings at 18 DPI (Table 2). The total percentage of seedlots transmitting compatible pathogens in symptomatic and epiphytic manner was 67%. None of the seedlings displayed symptoms for the seedlots which were generated from flowers inoculated with incompatible pathogens. In contrast, incompatible pathogens were detected as epiphytes on 19.4% (range: 4%–64.8%) (Table 2 and Table S1) of the seedlots tested, this type of transmission was termed as asymptomatic transmission in this study. With regards to epiphytic transmission, the percentage of positive seedlots were not significantly different for compatible and incompatible pathogens (*P* = 0.42). However, the mean percentage of positive seedlots for epiphytic transmission of null-interacting bacterial species (9.4; range: 6.09%–12.2%) (Table 2 and Table S1) was significantly lower (*P* = 0.001) than the seedlots for epiphytic transmission of compatible and incompatible pathogens. Irrespective of plant species, symptomatic transmissions of incompatible and null-interacting bacterial species to seedlings were not observed.

### Confirmation of bacterial strains re-isolated from infested seedlots (plating and SGO) by repetitive extragenic palindrome (rep)-PCR

DNA fingerprinting patterns obtained by rep-PCR of bacterial strains isolated from seedlots were identical to known patterns of respective reference strains used for comparison (data not shown). In addition, the isolated strains belonged to the same cluster as that of respective reference strains, which were used for flower inoculations.

### Temporal bacterial population dynamics on flowers of host and non-host plants

**Figure 1:**
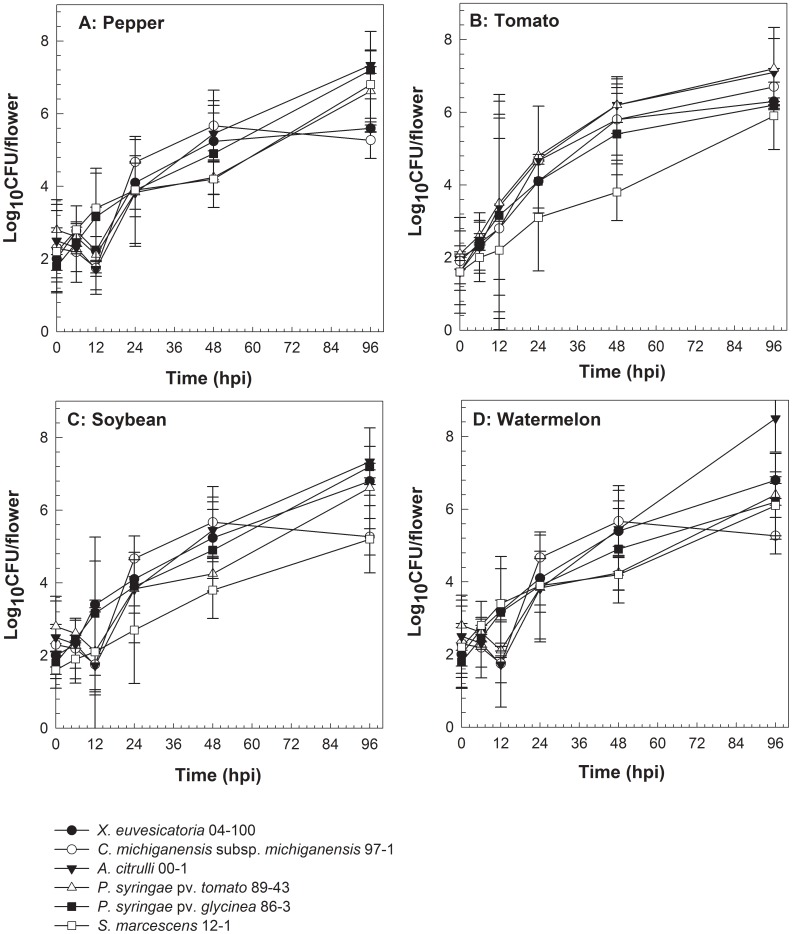
Temporal dynamics of Acidovorax citrulli, Clavibacter michiganensis subsp. michiganensis, Pseudomonas syringae pv. tomato, Xanthomonas euvesicatoria, Pseudomonas syringae pv. glycinea, and Serratia marcescens populations on pepper (A), tomato (B), soybean (C), and watermelon (D) flowers during 96 hour post inoculation (hpi). Three flowers per host/non-host-bacterial species combinations were sampled at 0, 6, 12, 24, 48, 96 hpi and populations of respective bacterial species were enumerated on semi-selective media. Data points represent the mean bacterial populations at each sampling period in two independent experiments. Bars indicate the standard error of the mean.

**Figure 2:**
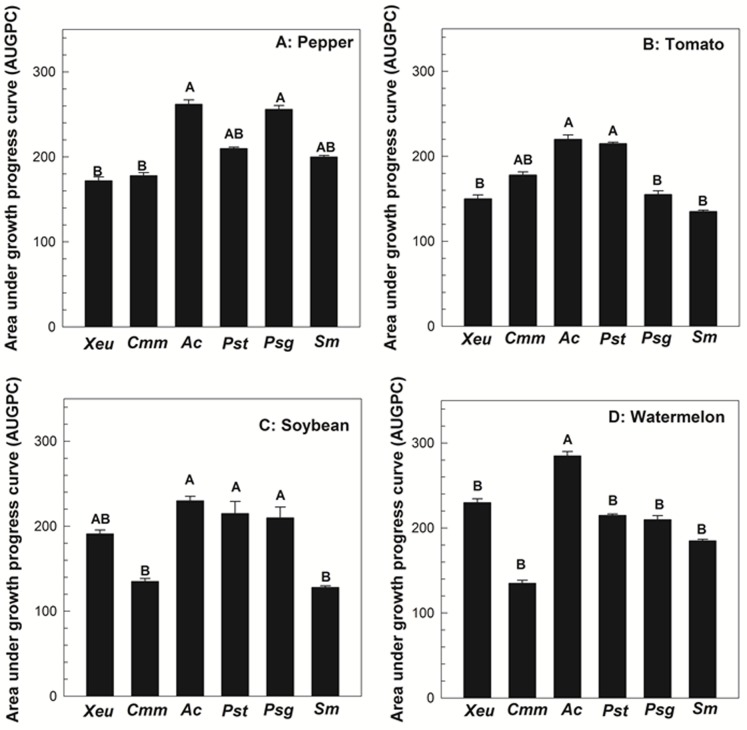
Bar chart of the area under the growth progress curves (AUGPC) calculated for Acidovorax citrulli (Ac), Clavibacter michiganensis subsp. michiganensis (Cmm), Pseudomonas syringae pv. tomato (Pst), Xanthomonas euvesicatoria (Xeu), Pseudomonas syringae pv. glycinea (Psg), and Serratia marcescens (Sm) populations on pepper (A), tomato (B), soybean (C), and watermelon (D) flowers during 96 hour post inoculation (hpi). Bars indicate the mean AUDPC values and lines indicate the standard errors of the means. Treatments with different letters are significantly different according to Fisher's test of least significant difference (P<0.05).

Target bacterial species were not recovered from the flowers (all tested plant species) whose stigmas were treated with PBS. On pepper flowers, the populations of *P. syringae* pv. *glycinea* (2.8×10^7^ CFU/flower), *A. citrulli* (5.7×10^7^ CFU/flower), *P. syringae* pv. *tomato* (7.2×10^6^ CFU/flower), and *S. marcescens* (3.7×10^6^ CFU/flower) increased exponentially by 96 hpi (Fig.1A). In contrast, during the same period, populations of *X. euvesicatoria* (2.5×10^5^ CFU/flower) and *C. michiganensis* subsp. *michiganensis* (6.2×10^5^ CFU/ blossom) increased to populations that were 10 to 100 fold lower than other tested bacterial species (Fig. 1A). Based on AUGPC values, significant differences were not observed among *P. syringae* pv. *glycinea*, *A. citrulli*, *P. syringae* pv. *tomato*, and *S. marcescens* strains (*P* = 0.271), respectively for their ability to colonize pepper blossoms (Fig. 2A). In contrast, AUGPC values for *X. euvesicatoria* and *C. michiganensis* subsp. *michiganensis* were significantly lower than *A. citrulli* and *P. syringae* pv. *glycinea* (*P*≤0.006) (Fig. 2A). However, the significant differences were not observed among *X. euvesicatoria*, C. *michiganensis* subsp. *michiganensis*, *P. syringae* pv. *tomato*, and *S. marcescens* (Fig. 2A).

On tomato flowers, exponential increase in *A. citrulli* (2.1×10^7^ CFU/flower), *P. syringae* pv. *tomato* (3.2×10^7^ CFU/flower), *C. michiganensis* subsp. *michiganensis* (6.2×10^6^ CFU/flower), *X. euvesicatoria* (5.3×10^6^ CFU/flower), and *P. syringae* pv. *glycinea* (4.2×10^6^ CFU/flower) populations were observed by 96 hpi (Fig. 1B). In contrast, approximately 100 fold difference in bacterial populations was observed for *S. marcescens* (3.2×10^5^ CFU/flower) (Fig. 1B). The AUGPC values for *A. citrulli* and *P. syringae* pv. *tomato* significantly higher than the AUGPC values for *X. euvesicatoria*, *P. syringae* pv. *glycinea* and *S. marcescens* (*P* = 0.004) (Fig. 2B). In contrast, the AUGPC value for *C. michiganensis* subsp. *michiganensis* was not significantly different with any of the bacterial species tested (Fig. 2B).

On soybean flowers, *X. euvesicatoria* (2.1×10^6 ^CFU/flower), *A. citrulli* (6.2×10^7^ CFU/flower), *P. syringae* pv. *tomato* (3.8×10^6^ CFU/flower), and *P. syringae* pv. *glycinea* (5.8×10^7^ CFU/flower) populations increased exponentially by 96 hpi (Fig. 1C). In contrast, *C. michiganensis* subsp. *michiganensis* and *S. marcescens* populations increased to 2.8×10^5^ CFU/flower and 1.8×10^5^ CFU/flower, respectively which were 10 to 100 fold lower than other bacterial species tested (Fig. 1C). The AUGPC values for *A. citrulli*, *P. syringae* pv. *tomato*, and *P. syringae* pv. *glycinea* were significantly higher than the AUGPC values for *C. michiganensis* subsp. *michiganensis* and *S. marcescens* (Fig. 2C). In addition, the AUGPC value for *X. euvesicatoria* in these tissues were not significantly different with any of the bacterial species tested (*P* = 0.019) (Fig. 2C).

On watermelon flowers, *A. citrulli* CFUs increased to 4.2×10^8^ CFU/flower whereas populations of *X. euvesicatoria* (1.5×10^6^ CFU/flower), *P. syringae* pv. *tomato* (1.3×10^6^ CFU/flower), *P. syringae* pv. *glycinea* (8.2×10^5^ CFU/flower), *C. michiganensis* subsp. *michiganensis* (2.2×10^5^ CFU/flower) and *S. marcescens* (6.4×10^5^ CFU/flower) remained 100 to 1000 fold lower than *A. citrulli* by 96 hpi (Fig. 1D). The AUGPC value for *A. citrulli* was significantly higher than other bacterial species tested (*P* = 0.003) (Fig. 2D). The AUGPC values for other bacterial species (except *A. citrulli*) were not significantly different (*P* = 0. 24) (Fig. 2D).

### Colonization of bacteria on host and non-host seeds

**Figure 3:**
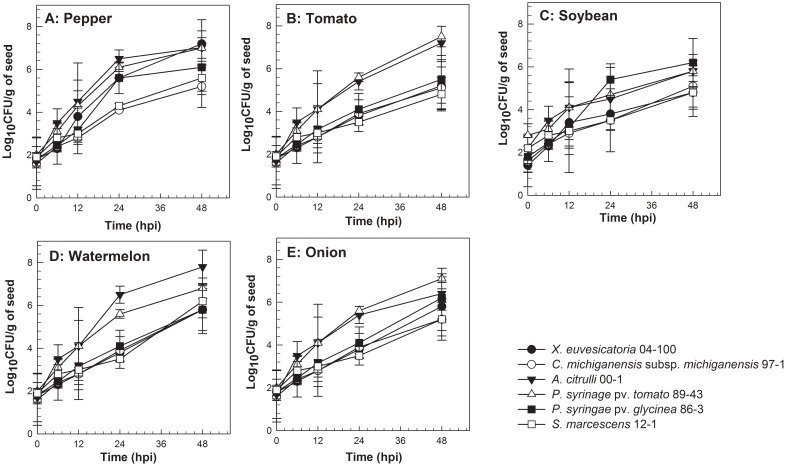
Temporal dynamics of Acidovorax citrulli, Clavibacter michiganensis subsp. michiganensis, Pseudomonas syringae pv. tomato, Xanthomonas euvesicatoria, Pseudomonas syringae pv. glycinea, and Serratia marcescens populations on pepper (A), tomato (B), soybean (C), watermelon (D), and onion (E) seeds during 48 hour post inoculation (hpi). Seed samples [(n = 1 g (pepper (150 seeds), soybean (20 seeds), watermelon (20 seeds), tomato (100 seeds), onion (200 seeds)] from three replicates per host/non-host-bacterial species combinations were sampled at 0, 6, 12, 24, 48, 96 hpi and populations of respective bacterial species were enumerated on semi-selective media. Data points represent the mean bacterial populations at each sampling period in two independent experiments. Bars indicate the standard error of the mean.

**Figure 4:**
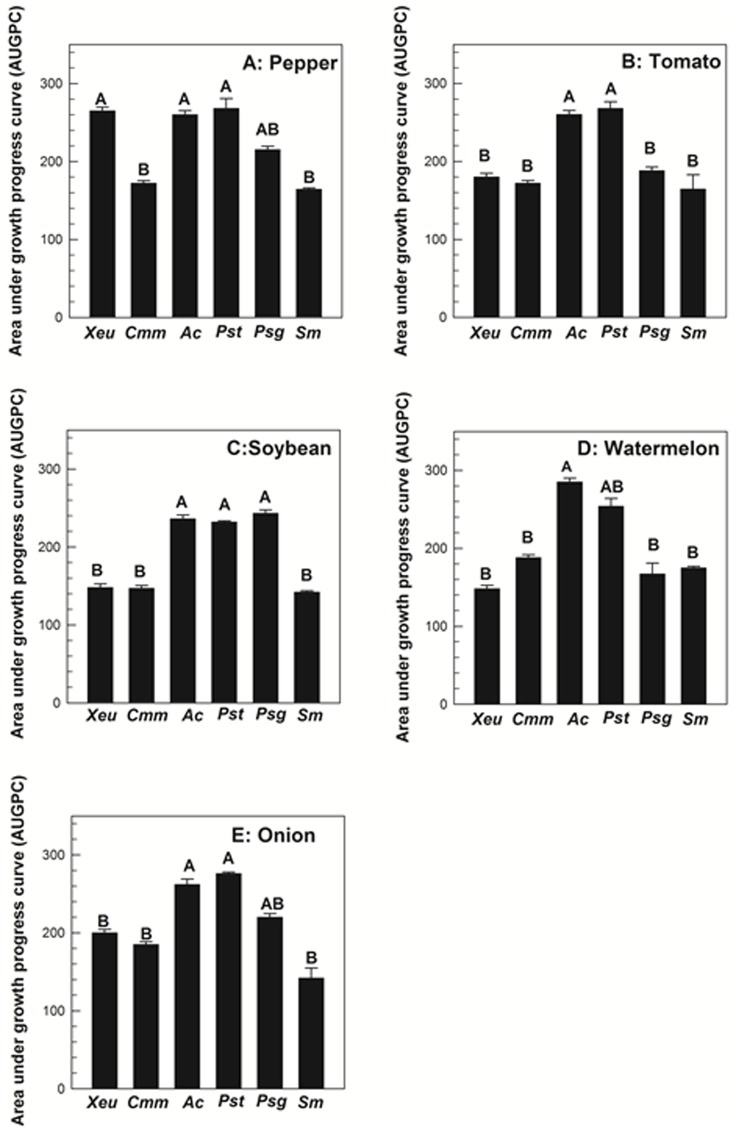
Bar chart of the area under the growth progress curves (AUGPC) calculated for Acidovorax citrulli (Ac), Clavibacter michiganensis subsp. michiganensis (Cmm), Pseudomonas syringae pv. tomato (Pst), Xanthomonas euvesicatoria (Xeu), Pseudomonas syringae pv. glycinea (Psg), and Serratia marcescens (Sm) populations on pepper (A), tomato (B), soybean (C), watermelon (D), and onion (E) seeds during 48 hour post inoculation (hpi). Bars indicate the mean AUDPC values and lines indicate the standard errors of the means. Treatments with different letters are significantly different according to Fisher's test of least significant difference (P<0.05).

Target bacterial colonies were not recovered from any of the seed types which were inoculated with PBS. Seed germination percentages were not affected for the treated seeds as they were not significantly different from negative controls (*P*≥0.72). On pepper seeds, CFUs of *X. euvesicatoria* (2.5×10^7 ^CFU/flower), *A. citrulli* (6.2×10^7^ CFU/flower), *P. syringae* pv. *tomato* (4.8×10^7^ CFU/g), and *P. syringae* pv. *glycinea* (1.8×10^6^ CFU/g) increased whereas populations of *C. michiganensis* subsp. *michiganensis* (3.8×10^5^ CFU/g) and *S. marcescens* (2.7×10^5^ CFU/g) remained 10 to 100 fold lower at 48 hpi (Fig 3A). In addition, *X. euvesicatoria*, *A. citrulli*, *P. syringae* pv. *tomato*, and *P. syringae* pv. *glycinea* displayed similar growth trends as their AUGPC values were not significantly different (*P* = 0.061); however, they were significantly higher than AUGPC values for *C. michiganensis* subsp. *michiganensis* and *S. marcescens* (*P* = 0.002) (Fig. 4A).

On tomato seeds, populations of *A. citrulli* and *P. syringae* pv. *tomato* increased to 7.2×10^7^ CFU/g and 6.4×10^7^ CFU/g, respectively by 48 hpi. During the same period, populations of *X. euvesicatoria* (2.1×10^5^ CFU/g), *P. syringae* pv. *glycinea* (3.2×10^5^ CFU/g), *C. michiganensis* subsp. *michiganensis* (2.7×10^5^ CFU/g), and *S. marcescens* (8.9×10^4^ CFU/g) increased exponentially but were 100-1000 fold lower than populations of *A. citrulli* and *P. syringae* pv. *tomato* (Fig 3B). Additionally, AUGPC values for *A. citrulli* and *P. syringae* pv. *tomato* were significantly higher than the AUGPC values of *X. euvesicatoria*, *P. syringae* pv. *glycinea*, *C. michiganensis* subsp. *michiganensis*, and *S. marcescens* (*P* = 0.002) (Fig. 4B). The AUGPC values for *X. euvesicatoria*, *P. syringae* pv. *glycinea*, *C. michiganensis* subsp. *michiganensis*, and *S. marcescens* were not significantly different from each other (*P* = 0.72) (Fig. 4B).

On soybean and onion seeds, populations of *A. citrulli* and *P. syringae* pv. *tomato* increased to ≥8.2×10^5^ CFU/g and ≥6.7×10^5^ CFU/g, respectively. The AUGPC values for both bacterial species were not significantly different on both seed types (*P*≥0.75) (Fig. 4C and E). On both seed types, populations of *P. syringae* pv. *glycinea* increased to the levels of 2.3×10^6^ (soybean) and 4.5×10^6^ (onion) CFU/g (Fig. 3C and E). The populations of *C. michiganensis* subsp. *michiganensis* [3.2×10^4^ CFU/g (soybean) and 4.8×10^4^ CFU/g (onion)] and *S. marcescens* [6.4×10^4^ CFU/g (soybean) and 1.3×10^4^ CFU/g (onion)] increased 100 fold from the initial populations of ∼10^2^ CFU/g on both seed types by 48 hpi (Fig. 3C and E). *Xanthomonas euvesicatoria* CFUs increased to 3.8×10^4^ CFU/g and 2.3×10^5^ CFU/g on soybean and onion seeds, respectively (Fig. 3C and E). On both seed types, significantly higher AUGPC values were observed for *A. citrulli* and *P. syringae* pv. *tomato* as compared to *X. euvesicatoria*, *C. michiganensis* subsp. *michiganensis*, and *S. marcescens* (*P* = 0.002) (Fig. 4C and E).

On watermelon seeds, *A. citrulli* CFUs increased exponentially and reached populations of 3.8×10^8^ CFU/g at 48 hpi (Fig. 3D). In contrast, populations of *X. euvesicatoria* (2.5×10^5^ CFU/g), *P. syringae* pv. *tomato* (3.6×10^6^ CFU/g), *P. syringae* pv. *glycinea* (4.2×10^5^ CFU/g), *C. michiganensis* subsp. *michiganensis* (5.7×10^5^ CFU/g), and *S. marcescens* (3.8×10^6^ CFU/g) remained 100 to 1000 fold lower than *A. citrulli* by 48 hpi (Fig. 3D). The population growth of *A. citrulli* was significantly higher than other bacterial species except *P. syringae* pv. *tomato*, based on AUGPC values (*P* = 0.004) (Fig. 4D). The trends of bacterial growth for *X. euvesicatoria*, *P. syringae* pv. *glycinea*, *P. syringae* pv. *glycinea*, *C. michiganensis* subsp. *michiganensis*, and *S. marcescens* were similar as AUGPC values for these bacterial species were not significantly different (*P* = 0.97) (Fig. 4D).

## Discussion

The infestation of seeds and subsequent transmission of pathogens has been well documented for many phytopathogenic bacteria with their hosts; however, studies on infestation and transmission of bacteria in non-host plants are limited. Previous studies showed that seeds of non-host plants can get infested with bacterial species and be transmitted to developing seedlings as epiphytes [7], [8]. This study provided the first evidence of saprophytic/epiphytic transmission of pathogens in non-host plants. In the current study, more detailed investigations on different parameters of seed infestation and transmission processes were evaluated with five seedborne phytopathogenic bacteria and a strain of non-pathogenic bacterium on five different plant species. The results from this study clearly demonstrated that seeds of non-host plants can be infested with seedborne phytopathogenic bacteria and subsequent transmission of pathogens can occur in an asymptomatic manner as epiphytes on non-host seedlings. Furthermore, results also indicated that flowers of non-host plants can be colonized by compatible/incompatible/null-interacting bacteria; however, the level of colonization differed significantly depending on the type of bacterial species used. Additionally, regardless of the type of bacterial interaction, bacterial species were also able colonize non-host seeds. However, bacterial population dynamics on seeds differed significantly depending on the type of bacteria tested. Similar observations of seed infestation, flower and seed colonization, and asymptomatic seedling transmission were also observed for a non-pathogenic strain of *S. marcescens* with the different plant species tested. Moreover, flower inoculations did not result in cross-contamination (in the greenhouse and field experiments) as the rep-PCR of bacterial strains isolated from seedlots displayed similar fingerprinting patterns to those of the reference strains, which were originally used for flower inoculation. In addition, none of the negative control seedlots were positive for the target bacterium by plating or SGO (symptomatic and asymptomatic transmission).

Irrespective of bacterial interaction with plant partner, inoculation of flowers resulted in the infestation of seeds. However, none of the fruits developed from inoculations with compatible/incompatible/null-interacting bacterial combinations displayed any symptoms. The percentage of seedlots infested with compatible and incompatible pathogens were significantly higher than with a null interacting pathogen, as determined by plating assay. These results indicated that compatible and incompatible interactions resulted in higher percentages of seed infestation as compared to a null-interaction. Darrasse *et al*. (2010) [7] determined the rate of seed transmission upon inoculation of bean flowers with *X. fuscans* subsp. *fuscans* CFBP4834-R or *X. campestris* pv. campestris ATCC 33913 or *E*. *coli* C600. Their results indicated that *X*. *fuscans* subsp. *fuscans* CFBP4834-R, being a compatible pathogen, can infest bean seeds with a higher frequency whereas the frequency of *X. campestris* pv. *campestris* ATCC 33913 (an incompatible pathogen) seed infestation was significantly lower. In addition, population densities of *X. campestris* pv. *campestris* ATCC 33913 on contaminated seeds was significantly lower than *X. fuscans* subsp. *fuscans* CFBP4834-R. In contrast, our results showed that the percentage of seed infestation did not differ significantly between compatible and incompatible interactions. The underlying reason for this observation is unclear; however, it is possible that the floral pathway of bacterial invasion to seed is permissive and is likely to be independent of any host-pathogen interactions. The permissive nature of the floral pathway could allow the contamination of seeds by a cohort of diverse bacteria, including bio-control agents [12], [41]. This hypothesis is supported by an observation made by Darsonval *et al*. (2008) [8] where Type III secretion system mutants of *X. citri* pv. *phaseoli* var *fuscans* were able to infest seeds through the floral pathway. In contrast, inoculation of these mutants through the plant vascular system did not result in seed infestation as observed for the wild-type strain. The authors concluded that a functional T3SS is not required for transmission to seeds in that particular incompatible interaction tested. In agreement to above study, Johnson *et al.* (2011) reported a type III secretion mutant of *A. citrulli* that retained the ability to colonize watermelon flowers and seeds similar to the wild-type strain [24].

Flower-inoculation with a null-interacting bacterial species (*S*. *marcescens*) also resulted in infestation of seedlots, regardless of the plant species tested. In contrast, previous studies reported that the inoculation of bean flowers with a null interacting pathogen e.g. *E. coli* did not result in the infestation of bean seeds. These results indicated that the infestation of seeds through the floral pathway is permissive to any bacterial species inoculated even with a null-interacting bacterium. Discrepancies were observed with null-interactions in previous observations and the present study, hence, a detailed investigation on seed infestation is required with number of null-interacting strains of *E. coli* and *S*. *marcescens* on different plant species.

Symptomatic seedling transmission of pathogens from infested seeds with compatible interactions has been observed for many phytobacterial pathosystem [5], [9], [10], [11], [20], [28], [45]. In addition, asymptomatic transmission of pathogens as epiphytes on a compatible host has been reported earlier [7], [8]. However, limited reports are available on asymptomatic seed-to-seedling transmission of bacterial species in incompatible and null-interaction situations. While seedlots (22.4%) infested with compatible pathogens developed symptoms by a SGO assay, seedlots generated by inoculation of incompatible and null-interacting pathogens remained asymptomatic. However, when seedlings from these lots were assessed for epiphytes, 19.4 and 9.4% of the lots were positive for respective incompatible and null-interacting bacterial species applied. In addition, lots which were asymptomatic by the SGO, when assessed for compatible pathogens growing as epiphytes on their respective hosts, 44.6% of the lots were positive. Seedlot infestation percentages were higher for lots with compatible bacteria than for incompatible and null-interacting bacteria as determined by the SGO (symptomatic and asymptomatic). These observations indicated that compatible, incompatible and null-interacting bacterial species can be transmitted as epiphytes on different plant species tested in the present study. However, the percentage of seedlots with epiphytes detected differed significantly based on the type of bacterial interaction with its plant partner. Darrasse *et al*. (2010) [7] reported epiphytic seed transmission and colonization of *X. campestris* pv. *campestris* ATCC 33913 on bean seedlings. Seeds developed from a flower inoculation of *X. campestris* pv. *campestris* ATCC 33913 did not develop symptoms on seedlings under favorable conditions. However, the pathogen was transmitted to growing bean seedlings as an epiphyte and bacterial populations of 1.9×10^5^ CFU/leaf disc were detected 4-day after planting. In our study, bacterial populations on host/non-host seedlings were not quantified rather qualitative assessments were made for the mere presence or absence of target bacterial species. We provided the first report of epiphytic transmission of the phytobacterial species *A. citrulli*, *C. michiganensis* subsp. *michiganensis*, *P. syringae* pv. *tomato*, *X. euvesicatoria*, *P. syringae* pv. *glycinea*, and *S. marecscens* on non-host seedlings. The reason behind the passage of bacterial species from seeds to seedlings is unclear. However, as far as epiphytic bacterial populations on non-host seedlings is concerned, previous reports indicate that bacterial seed-to-seedling transmission and subsequent colonization of seedlings rely mostly on surface colonization rather on parasitic relationships [7]. For compatible situations, seedborne bacteria colonize the surface of seedlings without any endophytic phase [15]. It is likely that such colonization behavior with seedborne bacteria in incompatible situations can also occur. Germinating seeds and developing seedlings exude copious amount of nutrients which may be utilized for seedborne bacterial colonization including in incompatible situation [32]. As such, authors concluded that bacteria may not require specific interactions with the plant to multiply efficiently in this environment. We did not employ the use of bacterial mutants containing defective genes for different pathogenicity determinants so similar comparisons with wild-type strains in incompatible and compatible situations could not be made.

Bacterial colonization of the flower (in particular stigma and style) is one of the most critical steps in seed infestation through the floral pathway [9], [11], [45]. In the present study, successful seed infestation was achieved by inoculating host/non-host flowers with 1×10^6^ CFU. However, it is unlikely that such a high inoculum level occur in natural environment especially in asymptomatic situations. Hence, for seed infestation via flowers to occur in nature, it is likely that bacteria will have to colonize floral tissues. To date, this aspect has been investigated only in compatible situations but not in incompatible and null-interaction scenarios. In the current study, the ability of different bacterial species to colonize floral tissues in compatible, incompatible and null-interactions were determined for 96-hpi. This study showed that bacteria can efficiently colonize the flowers of the different plant species tested, regardless of the interaction type. However, the population dynamics of bacterial species varied depending on the type of bacteria used. The dynamics of bacterial populations and associated AUGPC values for *A. citrulli*, *P. syringae* pv. *tomato*, and *P. syringae* pv. *glycinea* on host and non-host flowers were significantly higher as compared to other bacterial species. While *C. michiganensis* subsp. *michiganensis* colonization was significantly lower on non-host than on host flowers, *X. euvesicatoria* population dynamics was significantly lower on its host flower (pepper) as compared to other bacterial species (*A. citrulli* and *P. syringae* pv. *glycinea*). The behavior of *X. euvesicatoria* on pepper flowers as compared to other bacterial species is not well-understood. It is possible that the *X. euvesicatoria* strain or pepper cultivar used might have not supported the bacterial colonization on flowers as efficiently as with *A. citrulli* and *P. syringae* pv. *glycinea*. However, the success of *A. citrulli* and *P. syringae* pv. *glycinea* on pepper flowers indicated that the reason could be of bacterial origin. A null-interacting bacterium, *S. marcescens* could also colonize flowers of different plant species with bacterial populations reaching to ∼10^5^–10^6^ CFU/ flower by 96 hpi. Overall, these results indicate that flowers of host/non-host plants can be colonized by any bacterial species inoculated. It is likely that bacterial growth on the stigma can be supported by copious amount of stigma secretions (nutrients like carbohydrates, lipids and proteins) released during pollination. Previous reports of flower colonization of *A. citrulli*, *X. campestris* pv. *campestris* and *X. euvesicatoria* on watermelon, cauliflower and pepper, respectively showed that populations of these bacterial species can reach up to 1×10^5^ to 1×10^8^ CFU/flower by 96 hpi, when inoculated with 1×10^2^ CFU/flower without inciting disease symptoms [9], [11], [45]. In the present study, despite populations of all bacterial species on host/non-flowers reaching a level of ≥1×10^4^ CFU/flower within 96 hpi, disease symptoms or hypersensitivity reactions were never observed on developing fruits.

The ability of bacteria to colonize germinating seeds is an important step for pathogen transmission. For seed colonization, bacteria utilize nutrients in seed exudates released during germination [32], [34]. These seed exudates are rich in simple sugars, amino acids, organic acids, fatty acids and lipids which may provide necessary nutrients for bacterial establishment and colonization [32], [34]. Several reports on the bacterial population dynamics on host seed, during germination or early phase of seedling emergence, are available [32], [34]. However, few investigations have focused on the ability of bacterial pathogens to colonize seeds using incompatible or null-interactions. The present study assessed the ability of different bacterial species to colonize seeds in compatible, incompatible and null-interactions up to 48-h after inoculation. The results indicated that different bacterial species were able to colonize seeds of different plant species, irrespective of the type of interaction. However, population dynamics of bacterial species varied depending on the type of bacteria used. The population dynamics and AUGPC values for *A. citrulli*, *P. syringae* pv. *tomato*, and *P. syringae* pv. *glycinea* on host and non-host seeds were significantly higher than with other bacterial species. Additionally, *C. michiganensis* subsp. *michiganensis* and *S. marcescens* colonization of non-host seeds were significantly lower than with other bacterial species tested. Interestingly, *C. michiganensis* subsp. *michiganensis* seed colonization was significantly lower than *A. citrulli* and *P. syringae* pv. *tomato* on its own host seeds, tomato. The significantly reduced colonization of *C. michiganensis* subsp. *michiganensis* on tomato seed was surprising. It is possible that the strain used in this study might be less fit to grow on host/non-host seed. Future experiments with different strains of *C. michiganensis* subsp. *michiganensis* should be included to study the seed colonization ability of this bacterium.

Overall, to our knowledge, this is the first evidence of the seed infestation and asymptomatic transmission of seedborne bacterial pathogens resulting from flower inoculations of *A. citrulli*, *C. michiganensis* subsp. *michiganensis*, *P. syringae* pv. *tomato*, *X. euvesicatoria*, and *P. syringae* pv. *glycinea* on non-host seedlings, in this case pepper, tomato, onion, watermelon, and soybean. In addition, the inoculation of *S. marecscens* on flowers of non-host plants also resulted in infested seeds. Asymptomatic pathogen transmission as epiphytes was also observed on non-host seedlings. The outcomes from this study indicate that seeds of non-host plants can be infested with bacterial pathogens. Moreover, these bacterial pathogens can be transmitted to seedlings of non-host plants without inciting any symptoms, but rather as epiphytes thus indicating the probable role of non-host seeds as a primary inoculum source for bacterial pathogens. This research also provided clear evidence of the ability of these bacterial species to colonize floral tissues and seeds of host/non-host plants. The fact that *A. citrulli* could infest seeds of non-host plants and displayed higher colonization ability on the flowers and seeds of non-host plants relative to other bacterial species, worldwide spread of this pathogen in non-host seeds can be threatening. For an example, *A. citrulli* was reported to be seedborne and seed transmitted in tomato indicating that distribution of this pathogen can also occur in infested tomato seeds [1].

The four important parameters of successful seed-to-seedling transmission through the floral pathway (flower colonization, seed infestation, seed colonization and seedling transmission) have been successfully demonstrated in compatible, incompatible and null-interaction situations. These findings may have implications in the epidemiology, management, and regulatory issues of seedborne bacterial diseases. Bacterial pathogens transmitted on non-host seedlings as epiphytes, when planted in proximity to susceptible host seedlings may potentially disperse the pathogen resulting in unexpected epidemics. However, that step of bacterial dispersal from non-host seedlings to host seedlings has not yet been demonstrated. Future investigations on the potential dispersal of bacterial pathogens from non-host to host seedlings will be conducted. The implications on management of seedborne bacterial pathogens disseminating through non-host seeds may impact seed certification, seed quarantine, and seed health testing. Although, further investigation on the non-host seed/seedling transmission is required, data presented in this study suggest that seeds may potentially be a source of introduction of multiple bacterial pathogens.

## Conclusions

We provide the first evidence of the seed infestation and asymptomatic transmission of seedborne bacterial pathogens on non-host plants resulting from flower inoculations of *A. citrulli*, *C. michiganensis* subsp. *michiganensis*, *P. syringae* pv. *tomato*, *X. euvesicatoria*, and *P. syringae* pv. *glycinea* on tomato, watermelon, soybean, onion, and pepper. This research also provided clear evidence of the ability of these bacterial species to colonize floral tissues and seeds of non-host plants. We acknowledge that there is not only a risk of international movement of compatible seedborne pathogens but there may also be a potential risk of movement of incompatible plant pathogens through seedborne infestation via flower colonization. Bacterial pathogens transmitted on non-host seedlings as epiphytes, when planted in proximity to susceptible host seedlings may potentially disperse the pathogen resulting in unexpected epidemics. These findings may affect the management of seedborne bacterial pathogens disseminating through non-host seeds which may impact seed certification, seed quarantine and seed health testing.

## Supporting Information

Table S1 **Percentage of positive seedlots with compatible, incompatible and null-interacting bacterial species developed in fruits upon flower inoculation.**
(DOCX)
