# Supplementary material for: Interactions of Seedborne Bacterial Pathogens with Host and Non-Host Plants in Relation to Seed Infestation and Seedling Transmission
Source: PLoS One. 2014 Jun 17;9(6):e99215. doi: 10.1371/journal.pone.0099215 (PMC4061015; doi:10.1371/journal.pone.0099215)
Supplement: Table S1 — Percentage of positive seedlots with compatible, incompatible and null-interacting bacterial species developed in fruits upon flower inoculation. (DOCX) [file pone.0099215.s001.docx]

**Table S1**. Percentage of positive seedlots with compatible, incompatible and null-interacting bacterial species developed in fruits upon flower inoculation.

| Plant species | Bacteria | ^1^Interaction | Number of seedlots (*n*) | ^2^Seedlots positive by plating (%) | ^3^Seedlots positive by SGO (%), displayed symptoms | ^4^Seedlots with epiphytic bacterial population (%) |
| --- | --- | --- | --- | --- | --- | --- |
| Soybean (*Glycine max*) | *Xanthomonas euvesicatoria* | I | 74 | 40.5 | 0 | 4.0 |
|  | *Acidovorax citrulli* | I | 102 | 27.4 | 0 | 33.3 |
|  | *Pseudomonas syringae* pv. *tomato* | I | 82 | 24.4 | 0 | 32.9 |
|  | *Clavibacter michiganensis* subsp. *michiganensis* | I | 94 | 22.3 | 0 | 64.8 |
|  | *Pseudomonas syringae* pv. *glycinea* | C | 88 | 24.0 | 36.3 | 17.0 |
|  | *Serratia marcescens* | N | 82 | 18.3 | 0 | 12.2 |
| Watermelon (*Citrullus lanatus*) | *Xanthomonas euvesicatoria* | I | 68 | 66.2 | 0 | 26.4 |
|  | *Acidovorax citrulli* | C | 72 | 33.3 | 16.7 | 29.2 |
|  | *Pseudomonas syringae* pv. *tomato* | I | 47 | 17.0 | 0 | 19.2 |
|  | *Clavibacter michiganensis* subsp. *michiganensis* | I | 62 | 25.8 | 0 | 8.06 |
|  | *Pseudomonas syringae* pv. *glycinea* | I | 84 | 33.3 | 0 | 14.3 |
|  | *Serratia marcescens* | N | 72 | 27.8 | 0 | 11.1 |
| Tomato (*Solanum lycopersicum* L.) | *Xanthomonas euvesicatoria* | I | 86 | 39.5 | 0 | 5.82 |
|  | *Acidovorax citrulli* | I | 94 | 34.0 | 0 | 19.5 |
|  | *Pseudomonas syringae* pv. *tomato* | C | 108 | 33.3 | 16.7 | 32.4 |
|  | *Clavibacter michiganensis* subsp. *michiganensis* | C | 64 | 15.6 | 12.5 | 28.1 |
|  | *Pseudomonas syringae* pv. *glycinea* | I | 68 | 29.4 | 0 | 11.7 |
|  | *Serratia marcescens* | N | 87 | 13.8 | 0 | 8.04 |
| Pepper (*Capsicum annuum* L.) | *Xanthomonas euvesicatoria* | C | 84 | 52.3 | 29.8 | 26.2 |
|  | *Acidovorax citrulli* | I | 95 | 36.8 | 0 | 18.9 |
|  | *Pseudomonas syringae* pv. *tomato* | I | 64 | 18.8 | 0 | 9.38 |
|  | *Clavibacter michiganensis* subsp. *michiganensis* | I | 78 | 32.4 | 0 | 24.3 |
|  | *Pseudomonas syringae* pv. *glycinea* | I | 92 | 36.9 | 0 | 16.3 |
|  | *Serratia marcescens* | N | 83 | 14.5 | 0 | 9.63 |
| Onion (*Allium cepa*) | *Xanthomonas euvesicatoria* | I | 85 | 8.23 | 0 | 5.9 |
|  | *Acidovorax citrulli* | I | 78 | 26.4 | 0 | 13.5 |
|  | *Pseudomonas syringae* pv. *tomato* | I | 110 | 18.2 | 0 | 16.4 |
|  | *Clavibacter michiganensis* subsp. *michiganensis* | I | 66 | 51.5 | 0 | 31.8 |
|  | *Pseudomonas syringae* pv. *glycinea* | I | 72 | 29.2 | 0 | 11.1 |
|  | *Serratia marcescens* | N | 82 | 9.75 | 0 | 6.09 |

^1^ Type of interaction of bacterial species with their host and non-host plants. Abbreviations in the table I, C and N represent incompatible, compatible and null-interactions, respectively.

^2^Percentage of seedlots tested positive for compatible, incompatible and null-interacting bacterial species by plating. Means with similar letters are not significantly different according to LSD in SAS (*P* < 0.05).

^3^Percentage of seedlots tested positive by symptomatic transmission of compatible, incompatible and null-interacting bacterial species. A seedlot was considered to be positive when a seedling in a tray showed symptoms from any of the three replicates tested.

^4^Percentage of seedlots tested positive for asymptomatic (epiphytic) transmission of compatible, incompatible and null-interacting bacterial species to developing seedlings. A seedlot was considered to be positive when the target bacterium was recovered from any of the three replicates tested.
